# Isolation and characterization of fowl aviadenovirus serotype 11 from chickens with inclusion body hepatitis in Morocco

**DOI:** 10.1371/journal.pone.0227004

**Published:** 2019-12-31

**Authors:** Samira Abghour, Khalil Zro, Mohammed Mouahid, Fatima Tahiri, Meriam Tarta, Jaouad Berrada, Faouzi Kichou

**Affiliations:** 1 Division of Pharmacy and Veterinary Inputs, ONSSA, Rabat, Morocco; 2 Hassan 2^nd^ Institute of Agronomy and Veterinary Medicine, Rabat, Morocco; 3 Biopharma, Rabat, Morocco; 4 Mouahid’s Veterinary Clinic, Temara, Morocco; 5 Faculty of Sciences and Techniques Mohammedia, Hassan II University, Casablanca, Morocco; Panstwowy Instytut Weterynaryjny - Panstwowy Instytut Badawczy w Pulawach, POLAND

## Abstract

The present study was conducted in order to isolate, identify and characterize fowl aviadenovirus associated with inclusion body hepatitis (IBH) in three poultry farms (two of broiler chickens and one of breeder broiler chickens) in Morocco during 2015. Liver samples collected from affected three poultry farms were examined by histopathological examination. Tissue samples showing necrosis of hepatocytes associated with basophilic intranuclear inclusion bodies were homogenized and submitted to FAdV isolation in chicken embryo fibroblast (CEF) cell cultures and in SPF embryonated eggs. The cytopathic effect (CPE) was observed in the second passage with swelling and rounding of infected cells. The inoculated embryos were hemorrhagic and showed hepatitis with the presence of basophilic intra-nuclear inclusion bodies within hepatocytes. The presence of the virus was confirmed by conventional polymerase chain reaction based on hexon gene from all investigated samples. Moreover, phylogenetic analysis of the hexon gene revealed that FAdVs isolated from different affected poultry belonged to FAdV 11 serotype of the D genotype group. This work is the first isolation in cell culture and SPF embryonated eggs of FAdV from Moroccan broilers and breeder broiler chickens with IBH.

## Introduction

Inclusion body hepatitis (IBH) is an acute disease, mainly occuring in young broiler chickens (3–7 weeks old) and caused by several serotypes of fowl adenovirus (FAdV) [1]. FAdVs are classified within the *Aviadenovirus* genus, family of *Adenoviridae*, and are further classified into five species (FadV-A to FAdV-E) and 12 serotypes (FAdV-1 to 8a and 8b to 11) based on cross neutralization assay [2,3]. The IBH has been reported in other avian species, including turkeys [4], pigeons [5,6,7], geese [8], psittacine birds [9,10].

Natural outbreaks of IBH are characterized by a sudden onset of mortality which peaks within 3–4 days and return to normal by days 5–6. Mortality usually ranges from 5% to 10%, but can reach 30% [11,12]. The morbidity is low and sick chickens adopt a crouching position with ruffled feathers [6, 13]. FAdVs can be transmitted vertically through the embryonated egg or horizontally through feces, by means of personnel and transport [1].

At necropsy examination, the liver of affected birds is pale, friable, swollen and petechial hemorrhages may be present in skeletal muscle. Basophilic or eosinophilic intranuclear inclusion bodies are often observed in degenerated hepatocytes [13,14,15].

Diagnosis of IBH can be carried out through the observation of gross and histopathological changes, virus isolation and by polymerase chain reaction (PCR) allowing the detection of FAdVs with primer sequences based on the hexon gene [16,17,18]. DNA sequencing and restriction enzyme analysis can be used for FAdV typing [19,20].

In Morocco, the IBH was first reported in chickens between 2013 and 2015 based on necropsy and histopathology investigations. The lungs were congested and the livers were swollen, pale with necrosis of hepatocytes associated with large basophilic intranuclear inclusion bodies [21, 22]. In 2017, a case of IBH was described in broiler breeders [23]. Thereafter, some circulating FAdV from broiler poultry during 2017–2018 were identified and found to belong to FAdV-11 and FAdV-8a [24].

Despite the diagnosis of those cases, trials to isolate FadV have never been undertaken from field cases in Morocco. The aim of this work was to isolate and to characterize at the molecular level FAdV from field outbreaks of IBH that occurred during 2015. This can be used to implement a control strategy based on vaccination.

## Material and methods

### Ethics statement

This work was carried out in accordance with the guidelines of the National Food Safety Agency in Morocco (NFSAM). Embryonated eggs used were from the Division of Pharmacy and Veterinary Inputs (NFSA) which is reporting to the NFSAM.

Virus isolation was obtained from 17-day old SPF embryonated chicken eggs. Surviving embryos were anesthetized by intravenous injection in chorioallontoic vascular system using sodium pentobarbital (Dolethal, IPV Morocco) and all efforts were made to ovoid suffering.

### Samples collection and preparation

Liver samples were collected during 2015 from birds originating from 3 poultry farms (2 of broiler chickens and one of breeder broiler chicken) after being suspected at post-mortem examination to be affected by avian adenovirus infection. Fragments of liver tissues were fixed in 10% neutral buffered formalin (NBF) for histopathological examination. Other fragments of liver samples were aseptically collected in sterile bags and stored at -80°C until further processing. After thawing, the samples were homogenized in phosphate buffered saline (PBS) 10% containing 200U/ml penicillin and 0.2mg/ml streptomycin. Homogenates were centrifuged at 2000 g for 10 min at 4°C. The supernatant was filtered through 0, 45μm filter and transferred to fresh sterile tube and conserved at −80°C until further use.

### Histopathological examination

NBF-fixed tissues were embedded in paraffin according to standard methods, and 4 μm sections were performed, and stained with hematoxylin and eosin and examined under light microscope for microscopic changes.

### Preparation of chicken embryo fibroblasts cell culture (CEF)

Chicken embryo fibroblasts (CEF) cell culture was prepared from 11-day-old SPF chicken embryos according to standard procedure. The embryos were removed aseptically and washed twice with PBS. The embryos tissue was minced and washed gently with PBS and trypsinized gently with 0,25% trypsin-EDTA solution at 37°C for 20 min. The trypsinized cells were decanted and filtered through sterile gauze tied to sterile beaker. To stop the activity of residual trypsin, the growth medium (Eagle’s medium) (MEM) supplemented with 10% foetal calf serum (FCS), 100U/ml penicillin and 0.1 mg/ml streptomycin. The filtrate containing cells was centrifuged at 1500 rpm for 20 min at 4°C. The cell concentration was adjusted to 5 x 10^6^ cell/ml of the medium and cell suspension in 10ml volume was seeded in 25 cm^2^ tissue culture flasks and incubated at 37°C under 5% CO2 until confluent monolayer is formed.

### Isolation of FAdV in CEF cell culture

The complete CEF monolayer cultures were washed with PBS and 500 μl of liver tissue homogenates were inoculated in 25 cm^2^ tissue culture flasks. The inoculum was allowed to adsorb onto the cells at 37°C for 60 min, after that, 8ml of the maintenance medium containing 2% FCS was added to the culture. The infected flasks and uninfected flaks used as control, were incubated at 37°C under 5% CO2 and the monolayers were observed for cytopathic effect (CPE) daily for 7days. Inoculated cells were passaged by three cycles of freezing and thawing the flasks, then centrifuged at 2000 rpm for 10min. 500 μl of supernatant was inoculated onto freshly prepared CEFs cell monolayers.

### Isolation of FAdV in SPF embryonated chicken eggs

Isolation was also attempted with 10-day old SPF embryonated chicken eggs (ECEs) by inoculating 0,1ml of liver tissue homogenates by the chorioallontoic route into five ECEs per sample. Five ECEs inoculated with phosphate buffer saline (PBS) were used as a negative control. The eggs were incubated at 37°C for 7 days and candled daily. Embryos mortalities which occurred before 48h were regarded as non-specific and discarded. All embryos which died after 48h post-inoculation, as well as all those which survived until experiment termination, were harvested and necropsied. Liver samples were collected for histopathological and molecular examinations.

### FAdV detection by PCR

#### DNA extraction

Aliquots (200 μl) of supernatant from second and third passage of infected cell monolayers as well as the supernatant of homogenized liver tissues of affected chickens and inoculated embryos were processed for extraction of viral DNA using the Macherey Nagel Kit (Nucleospin Tissue, Germany) according to the manufacturer’s instructions but without preparing and pre-lyse sample steps. Briefly, the supernatant was mixed with 200μl and 210 μl of buffer B3 and 100% ethanol respectively. DNA was eluted in 100μl of nuclease-free water, and 5μl per PCR was used for the template.

#### Primers set

The primers were designed from conserved reported sequences identical to a region of the hexon protein gene of several FAdVs (group I–III) that can be used to identify the group and type. The specificity of the following primers has been widely tested in previous studies: HexF1, 5’GAYRGYHGGRTNBTGGAYATGGG-3’ and HexR1, 5’-TACTTATCNACRGCYTGRTTCCA-3’ [16]. These primers theoretically yield an 800 bp amplicon.

#### Polymerase chain reaction (PCR)

Reagents from Taq DNA Polymerase recombinant kit (Invitrogen, ThermoFisher, USA) were used to prepare the reaction mix. PCR was performed in a final volume of 25μl containing 2.5μl of 10X Taq polymerase buffer, 2 mM magnesium chloride, 0.8 mM dNTP Mix and 2.5 U of Taq DNA polymerase. PCR was amplified using the following cycling profile: one initial denaturation step at 95°C for 5 min; 35 cycles of 94°C for 30 s, 55°C for 30 s, and extension at 72°C for 30 s. The liver sample from clinical affected chickens confirmed positive by histological examination was used as a positive control whereas DNA extracted from SPF poultry liver was used as a negative control.

#### Agarose gel analysis of PCR products

Agarose gel electrophoresis (1.2% UltraPureTM Agarose, Invitrogen, USA) was used to analyze the amplified DNA. The DNA fragments were visualized by Omega Lum G instrument (Aplegen, USA).

### FAdV sequencing and phylogenetic analysis

The amplified PCR fragments were purified using EXOSAP-IT (USB, USA) and bidirectionally sequenced on an ABI 3130 Xl automated sequencer (Applied Biosystems, Foster City, CA, USA) using BigDye Terminator version 3.1 Kits with the same primers as those used for the amplification. The analysis of electrophoregram was done using the sequencing Analysis Software version 5.3.1 (Applied Biosystems, Foster City, CA, USA). The sequences were first aligned by using the Clustal W program. The FAdV sequences were edited and compared with published sequences available in GenBank, using BLAST tool of the National Center for Biotechnology Information NCBI. Evolutionary trees for the data set were inferred by using the Neighbor-Joining program of MEGA version 6. The stability of relationships was assessed by performing bootstrap analyses of the Neighbor-Joining data based on 500 resamplings. The FAdV sequences generated have been deposited in the GenBank database under the accession numbers MK468898, MK468899 and MK468900.

## Results

### Histopathological findings

**Fig 1:**
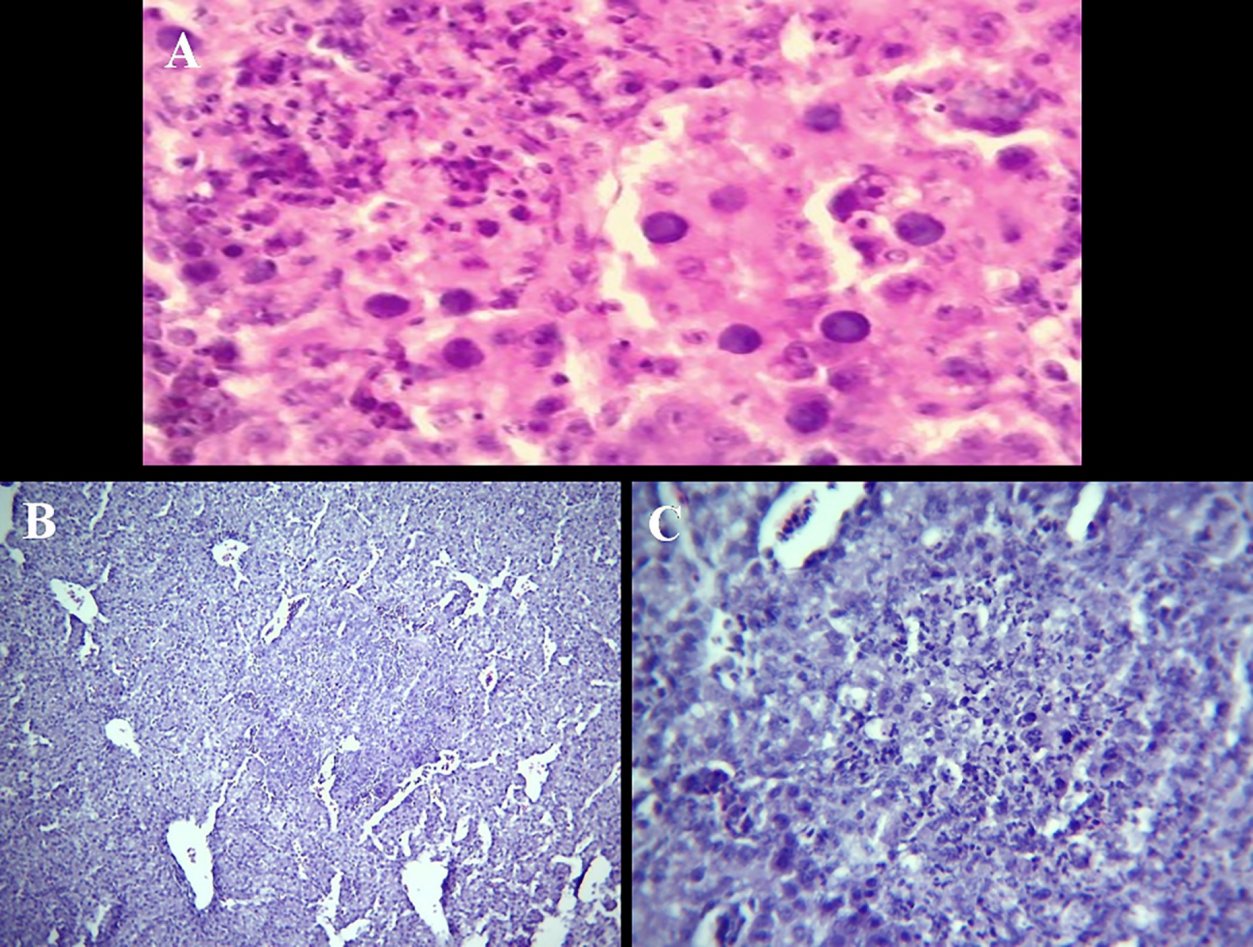
**A.** Liver with very severe hyperemia, severe necrosis of individual/groups of hepatocytes and the presence of basophilic intra-nuclear inclusion bodies within hepatocytes. H&E, X400. **B**. Liver of SPF- chicken embryo inoculated with field liver homogenates showing multifocal necrosis of groups of hepatocytes and the presence of basophilic inclusions bodies (intra-nuclear) within hepatocytes. H&E, X40. **C**. Liver of SPF chicken embryo (higher magnification of Fig B)–foci of hepatocytes necrosis. Cellular debris and basophilic intra-nuclear inclusion bodies. H&E, X160.

Histopthological examination of liver originating from the 3 affected chicken farms revealed severe and extensive hepatic necrosis associated with the presence of basophilic intra-nuclear inclusion bodies within hepatocytes compatible with the diagnosis of inclusion body hepatitis (Fig 1).

### Isolation of the virus in CEF cell culture

**Fig 2:**
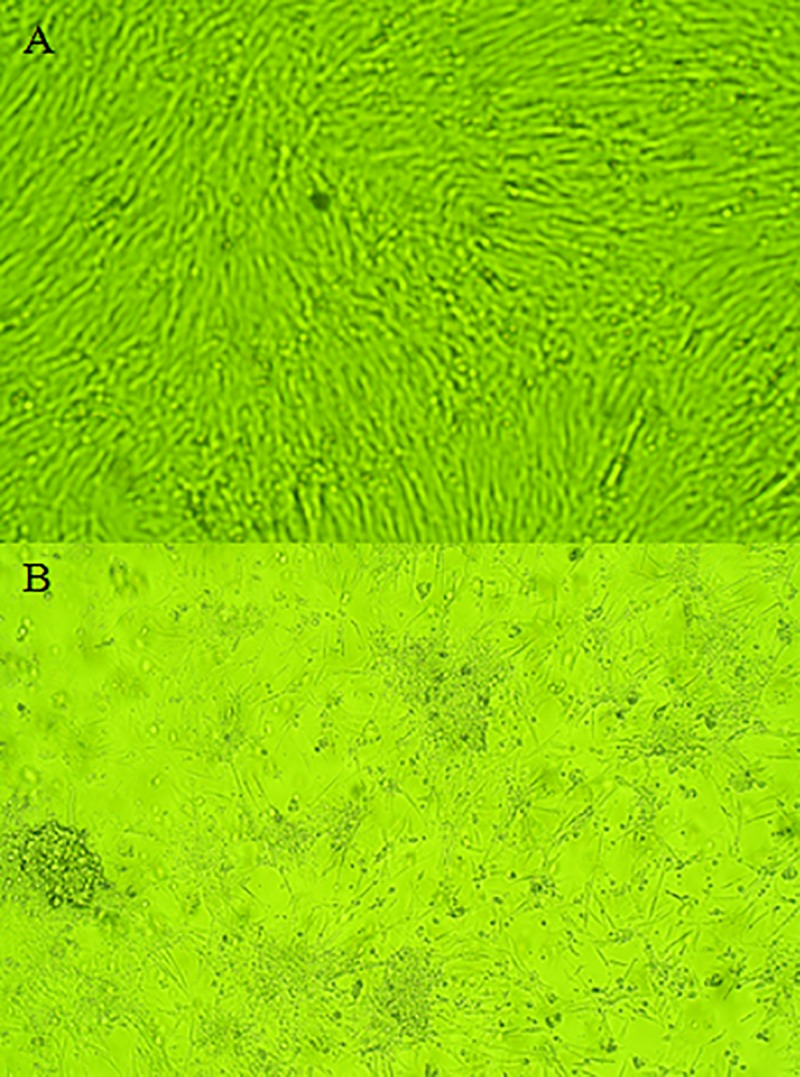
Cytopathic effect of FAdV in CEF cells. A) Eighty percent confluent CEF cells. B) Typical cytopathic effects shown as cell rounding, refractility and detachment.

Monolayers of CEF cultures were infected with liver homogenates of affected chickens from the three farms. The first CPE was observed at about 48 h post-inoculation during the second passage. Infected cells were rounding and started to detach from the monolayers and clump by 72h whereas no such changes were observed in the uninfected CEFs cell cultures. The third passage gave CPE within 24h post-infection and involved majority of the cells in monolayers. Micro-photographies of uninfected chicken embryo fibroblasts cell culture and monolayer of CEF cell culture showing CPE are given in (Fig 2).

### Isolation of FAdV in SPF embryonated chicken eggs

**Fig 3:**
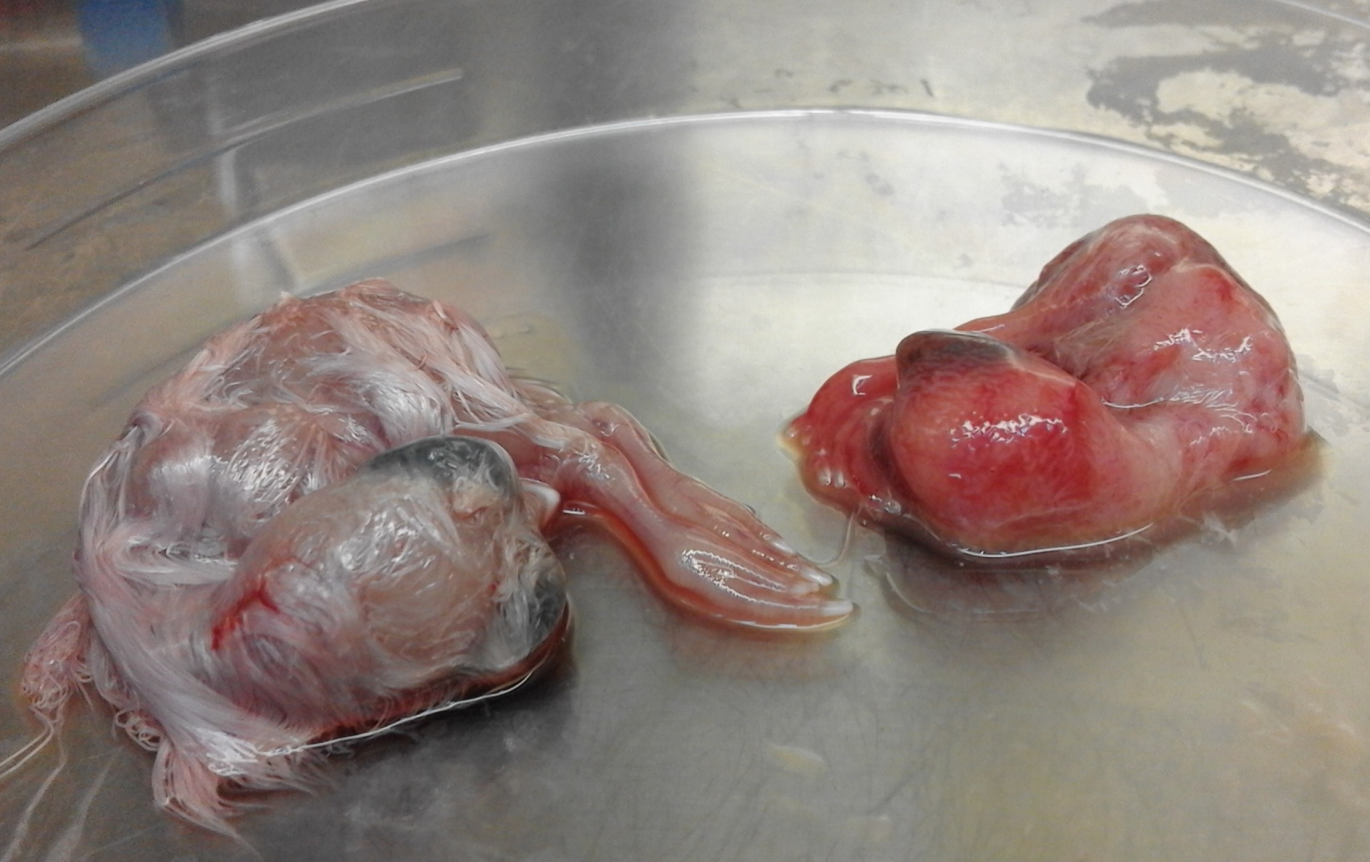
Isolation of FAdV using embryonated chicken eggs. An infected embryo shows curling and dwarfing 4 days after inoculation in comparison to the control embryo.

**Fig 4:**
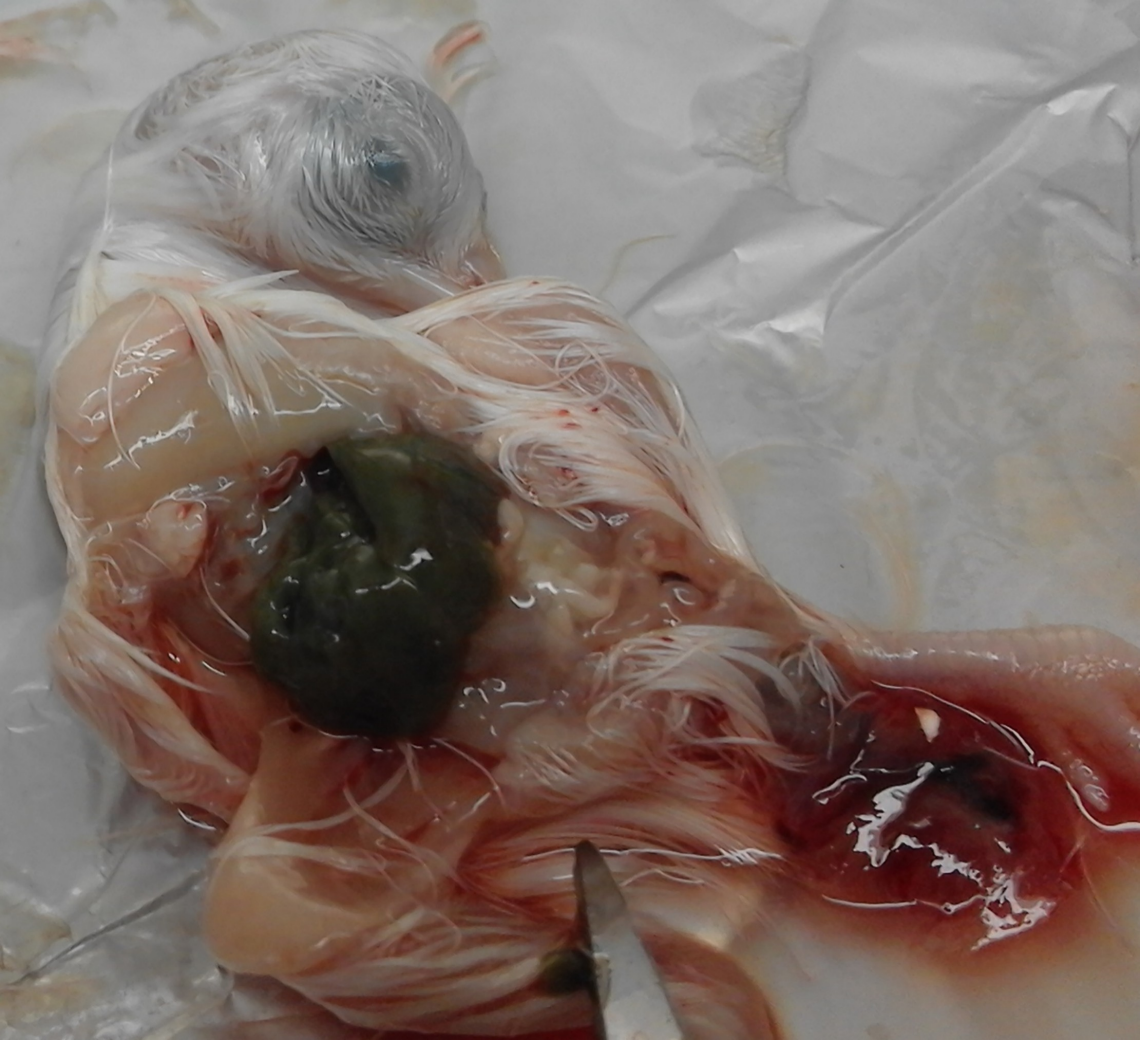
Inoculated embryo with liver enlarged and showed a diffuse greenish discolouration.

All embryos inoculated with liver homogenate of affected chickens from the three poultry farms, died after 3–4 days post inoculation. The inoculated embryos were hemorrhagic, and showed enlarged livers, with either yellow to reddish foci or diffuse greenish discoloration (Figs 3 and 4). Histological examination showed acute hepatitis with necrotic hepatocytes and presence of basophilic intra-nuclear inclusion bodies within hepatocytes.

### Virus detection by PCR

**Fig 5:**
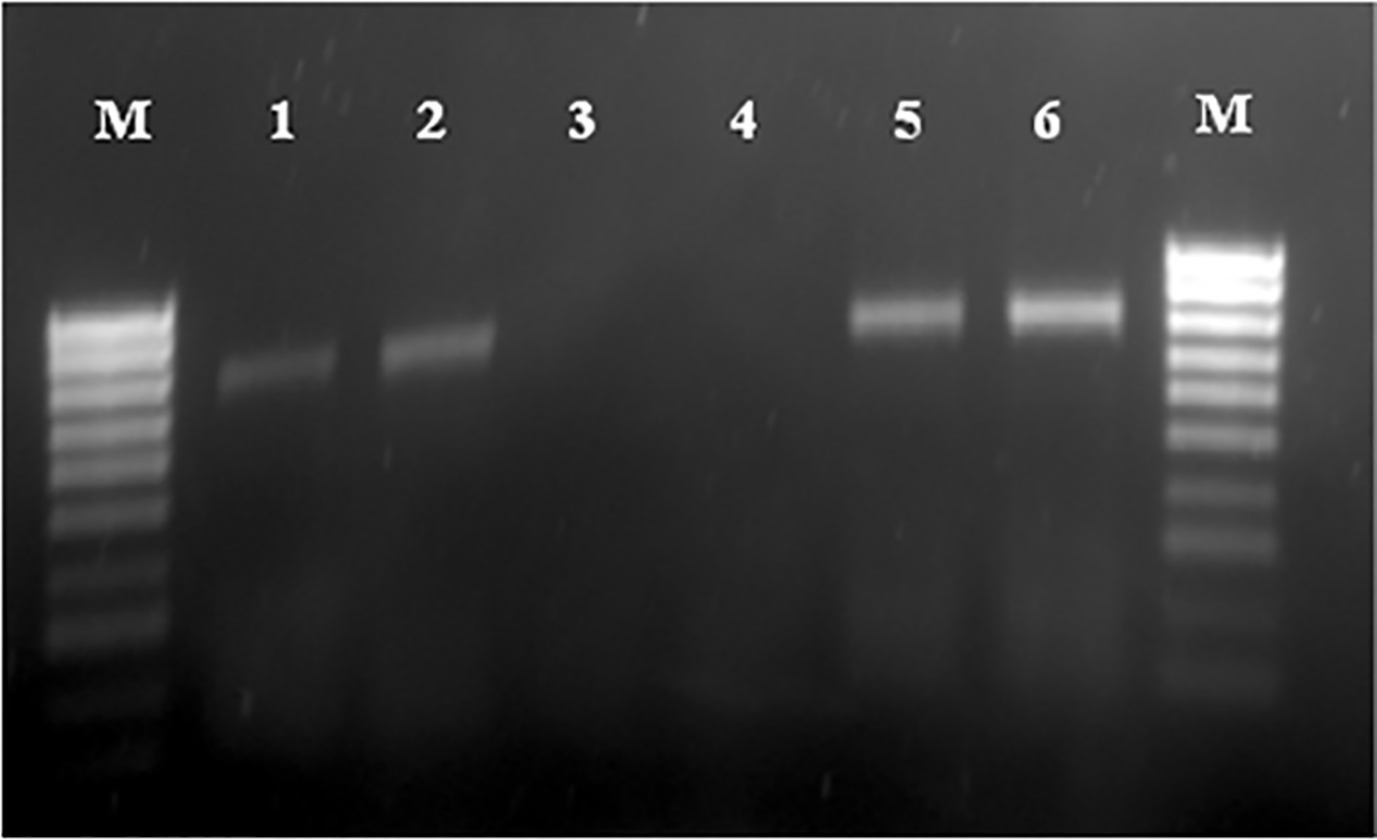
Hexon gene DNAs of fowl aviadenovirus detected from samples, Lane 1: Homogenized liver tissues (Positive control). Lane 2: Inoculated embryos. Lane 3: Extraction negative control. Lane 4: Amplification negative control. Lane 5: Supernatant of second passage of infected cell culture. Lane 6: Supernatant from third passage of infected cell culture. Lane M: Molecular weight 100 pb.

The PCR products visualized in agarose gel electrophoresis showed the presence of amplified DNA products of 800 bp in all the DNA samples from the supernatant of second and third passages of infected CEF cells and from liver homogenates of affected chickens of the three poultry farms as well as from liver homogenates of inoculated embryos (Fig 5). This result confirms the presence of hexon protein gene specific for fowl adenovirus.

These molecular investigations have confirmed that Fowl adenovirus is causative agent of IBH in broiler and broiler breeders chickens in Morocco.

### Sequence alignment and phylogenetic analysis

**Fig 6:**
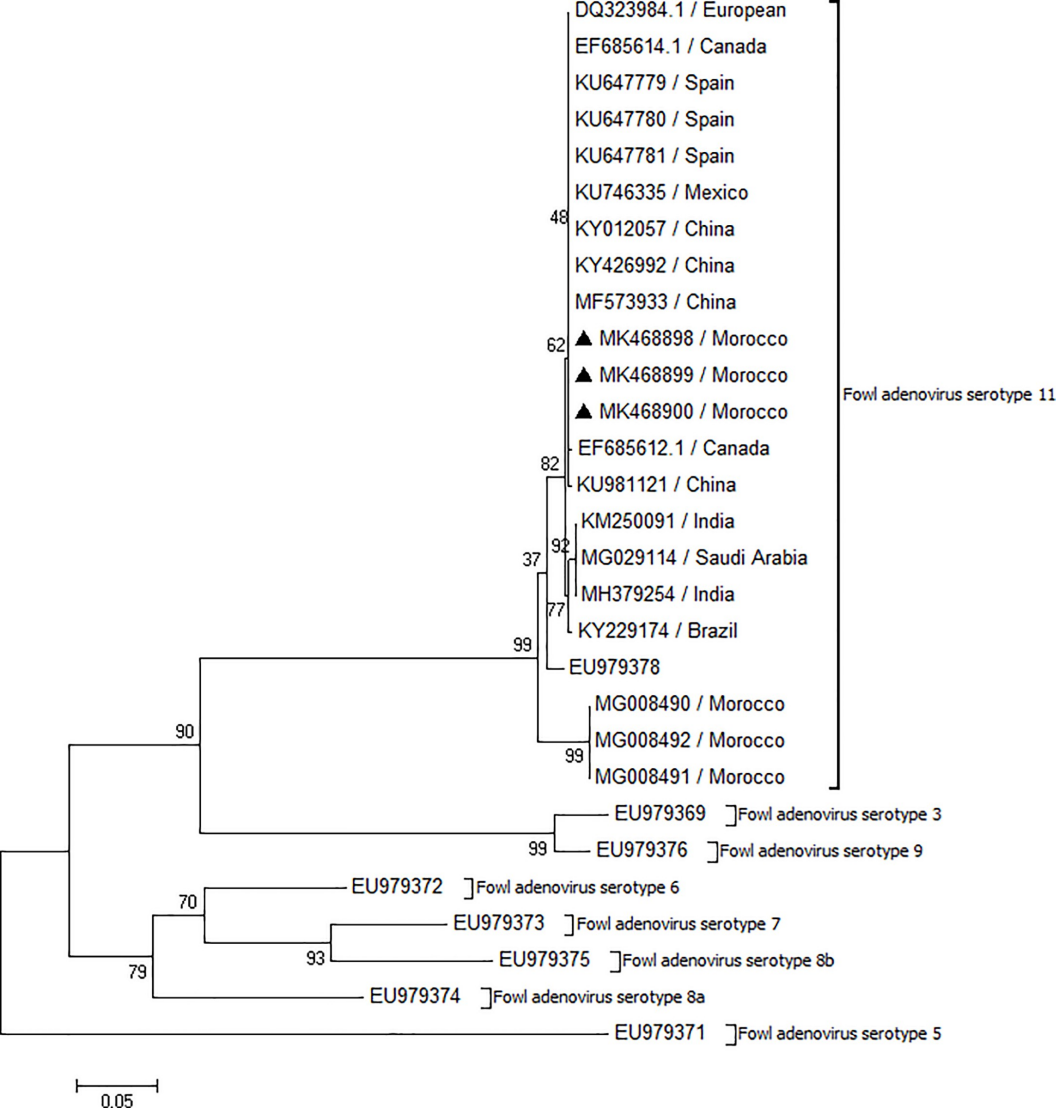
Phylogenetic tree based on nucleotide sequence of hexon gene of FAdV field isolates and sequences from gene bank.

The nucleotide sequences of the three FAdV strains isolated in Morocco in both broiler and broiler breeder farms were completely identical (100%). According to phylogenetic analysis based on the hexon gene with available sequences from GenBank, all FAdVs were classified as FAdV-D serotype 11 (Fig 6), showing the same nucleotide identities with the European serotype 11 isolated also in USA (Accession n° DQ323984) and Chinese serotypes (Accession n° MF 573933, KY 012057).

Interestingly, maximum similarities (99%) with the other isolated strains in India and Saudi Arabia have been noted.

We also compared our nucleotide sequences of the three FAdV strain with the sequences that were identified in Morocco in 2018 by Redondo et al, the result showed a sequence identity of 94%.

On the other hand, the nucleotide sequences of Moroccan FAdVs strains showed low sequence identity (<80%) with Fowl adenovirus 11- UF71 strain (Accession n° EU979378) and C2B strain (Accession n° AF508959).

## Discussion

In recent years, the clinical cases of IBH have been increasing all over the world [25], resulting in considerable economic losses in many countries, such as USA [26], India [27], Canada [28], Hungary [29], Korea [30], Lebanon [31] and Spain [32]. Most of these outbreaks have been associated with genotypes FAdv-D or–E, with serotypes 2, 11, 8a and 8b being the most frequently involved [25].

In Morocco, outbreaks of IBH in chickens were reported in 2013 [21]. Diagnosis was based on postmortem and histopathological examinations which revealed enlarged and pale liver with the presence of basophilic intranuclear inclusion bodies in hepatocytes. Thereafter, several other cases of IBH in 2 to 3 wk-old broiler chickens were detected on 2015 based on macroscopic and microscopic changes [22] followed by one case of IBH that has been described in broiler breeders in 2017 [23]. In 2018, the FAdV from broiler poultry was characterized and found belonging to FAdV-11 and FAdV-8a [24]. In most of the cases of IBH in Morocco, the FAdV was never been isolated and identified as the causative agent of IBH. In the present study two broiler farms and one broiler breeder farm affected by IBH during 2015 were investigated in order to determine and confirm the causative agent. The liver samples of affected chickens from the three farms were examined by histopathology, and the FAdV was successfully isolated in chicken embryo fibroblasts (CEF) cell culture and in SPF embryonated eggs. Then the FAdV was identified by conventional PCR based on hexon gene and characterized by phylogenetic assays.

The histopathological findings in livers from naturally affected chickens (of the three farms) and inoculated embryos including necrosis of hepatocytes with basophilic intranuclear inclusion bodies (INIB) are similar to the observations of earlier workers [33, 34, 35]. The presence of intranuclear inclusion bodies in the hepatocytes is considered typical of FAdV infection in IBH. Indeed, the presence of INIB in hepatocytes have been shown by inoculation of FAdV to specific pathogen free (SPF) embryonated eggs via the chorioallantoic membrane and was observed at 5–6 dpi and the adenovirus particles were identified in liver samples of embryos by transmission electron microscopy [36]. Furthermore, SPF chickens infected with different strains of FAdV showed the presence of INIB in hepatocytes at the histological examination of liver tissues [32, 37, 38].

The FAdV was isolated in chicken embryo fibroblast (CEF) cell culture inoculated with liver tissue homogenates. The cytopathic effect (CPE) was observed in the second passage. Swelling and rounding of the infected cells appeared within 48h of infection, and by 72h the cells started detaching from the monolayers. In earlier studies in which the CEF cell cultures were inoculated with different strain of FAdV, the CPE was observed in second or third passage [39, 40, 41]. The isolation of FAdV can be performed in different types of cell cultures, therefore in the majority of studies, the virus isolation of FAdV is performed on primary chicken embryo liver (CEL) cells [17,35,42,43,44,45,46,47,48]. In other studies, the FAdV isolation was performed in chicken embryo kidney (CEK) cell cultures [49,50,51]. The isolation of FAdV was successful in chicken hepatoma (CH) cell line as well [52, 53, 54]. In the present work FAdV was also isolated in SPF embryonated chicken eggs from liver homogenates. The inoculated embryos were hemorrhagic and livers were enlarged with yellow, reddish foci and/or diffuse greenish discoloration. The histopathological examination of liver samples of inoculated embryos showed necrotic hepatocytes with presence of basophilic intra-nuclear inclusion bodies within hepatocytes. This result confirms the presence of FAdV in embyonated eggs. These results were similar to those reported by earlier workers [42,55]. In the present study, FAdV was successfully isolated in CEF cell cultures and in SPF embryonated eggs from the liver samples collected from affected chickens of the 3 flocks.

The molecular tool of PCR is being widely used to amplify and detect FAdVs specific gene in clinical samples for confirmatory diagnosis of diseases associated with these viruses [56, 57]. In our study the cell culture supernatant from second and third passage of the three isolates as well as the liver homogenates of affected chickens and liver homogenates of inoculated embryos were subjected to DNA extraction and PCR amplification using primer pair HexF1/HexR1. Specific product of 800 bp was detected by electrophoresis in 1% agarose gel. These results confirm the presence of FAdV in the infected cell culture fluid, in liver samples of the three poultry farms and in inoculated embryos. In this study we used the same primers used by earlier workers [16, 49, 58].

The nucleotide sequences analysis showed that the three FAdV isolates belonged to FAdV serotype 11 of the D genotype group. This strain was responsible for different inclusion body hepatitis outbreaks in poultry in Canada [28], China [59], Hungary [29], Australia [38], Lebanon [31], Spain [32] and Iran [60].

In phylogenetic analysis, the sequences of three FAdV strains were compared with the sequences identified in Morocco in 2018 by Redondo et al. The result showed a sequence identity of 94%.

The phylogenetic analysis revealed that the three FAdVs strains have the same nucleotide identities with the European serotype 11. This result suggests that the FAdVs isolated were most probably introduced into the country through vertically infected day old broiler breeder birds imported from Europe.

The high similarity between breeder and broiler FAdV suggests that the viruses may vertically transfer to the progeny and spread laterally among the flocks.

The recent work, Redondo et al. (2018) has identified FAdV 11 and 8b in broiler chickens in Morocco while Salek and El Houadfi described a case of IBH in broiler breeders chicken with FAdV 11 in 2017. However, IBH virus isolation has never been carried out in Morocco. Virus isolation is a crucial step in vaccine development and control strategies of IBH in the country. In the present work, FAdV was successfully isolated in CEF cell culture and in SPF embryonated eggs from liver samples of broilers and broiler breeders with IBH.

To our knowledge, this is the first work in which the isolation and identification of Moroccan FAdV in CEF cell culture and in SPF embryonated eggs were achieved from broiler and broiler breeder poultry with IBH. Phylogenetic analysis of the FAdV hexon gene of the isolated virus proved that it closely related to a strain previously classified as serotype FAdV 11. These results are the starting point for further investigations into the epidemiology of FAdVs in Morocco and for pathogenicity studies of the local isolated viruses.

## References

[1] McFerran JB, Adair BM. Group I adenovirus infection In: Saif YM, Barnes HJ, Glisson JR, Fadly AM, McDougald LR et al, editors. Diseases of poultry, 11th Ed Ames: Iowa State Press 2003; pp. 214–227.

[2] Benko M, Harrach B, Russel WC. Family Adenoviridae In: van Regenmortel MHV et al, editors. Virus Taxonomy. Classification and nomenclature of viruses. Seventh Report of the International Committee on Taxonomy of Viruses: Academic Press, San Diego; 2000 pp. 227–238.

[3] Hess M. Detection and differentiation of avian adenoviruses: A review. Avian Pathol. 2000; 29: 195–206. 10.1080/03079450050045440 19184805

[4] Guy JS, Schaeffer JL, Barnes HJ. Inclusion-body hepatitis in day-old turkeys. Avian Dis. 1988; 32: 587–590. 2848488

[5] Hess M, Prusas C, Monreal G. Growth analysis of adenoviruses isolated from pigeons in chicken cells and serological characterization of the isolates. Avian Pathol. 1998; 27: 196–199. 10.1080/03079459808419323 18483986

[6] Mc Ferran JB, McCracken RM, Connor TJ, Evans RT. Isolation of viruses from clinical outbreaks of inclusion body hepatitis. Avian Pathol. 1976; 5: 315–324. 10.1080/03079457608418201 18777361

[7] Takase K, Yoshinaga N, Egashira T, Uchimura T, Yamamoto M. Avian adenovirus isolated from pigeons affected with inclusion body hepatitis. Japanese Journal of Veterinary Science. 1990; 52: 207–215. 10.1292/jvms1939.52.207 2161475

[8] Riddell C. Viral hepatitis in domestic gees in Saskatchewan. Avian Dis. 1984; 28: 774–782. 6091609

[9] Capua I, Liberti L, Gough RE, Casaccia C, Asdrubali G. Isolation and characterization of an adenovirus associated with inclusion body hepatitis in psittacine birds. Avian Pathol. 1995; 24: 717–722. 10.1080/03079459508419110 18645827

[10] Droual R, Woolcock PR, Nordhausen RW, Fitzgerald SD. Inclusion body hepatitis and hemorrhagic enteritis in two African grey parrots (Psittacus erithacus) associated with adeno-virus. J. Vet. Diagn. Invest. 1995; 7: 150–154. 10.1177/104063879500700125 7779952

[11] Hess M. Aviadenovirus infections In: Swayne D, Glisson JR, McDougald LR, Nolan LK, Suarez DL, V, editors. Diseases of poultry, 13th ed Ames: Wiley-Blackwell 2013; pp. 290–300.

[12] Schachner A, Marek A, Grafl B, Hess M. Detailed molecular analyses of the hexon loop1 and fibers of fowl aviadenoviruses reveal new insights into the antigenic relationship and confirm that specific genotype are involved in fied outbreaks of inclusion body hepatitis. Vet Microbiol. 2016; 186: 13–20. 10.1016/j.vetmic.2016.02.008 27016752

[13] McFerran JB, Smyth JA. Avian adenoviruses. Rev.—Off. Int. Epizoot. 2000; 589–601. 10935281

[14] Grimes TM, King DJ, Kleven SH, Fletcher OJ. Involvement of type-8 avian adenovirus in the etiology of inclusion body hepatitis. Avian Dis. 1977; 21, 26–38. 190994

[15] Hair-Bejo M. Inclusion body hepatitis in a flock of commercial broiler chickens. J. Vet. Malaysia.2005; 17: 23–26.

[16] Mase M, Mitake H, Inoue T, Imada T. Identification of group I-III avian adenovirus by PCR coupled with direct sequencing of hexon gene. J. Vet. Med. Sci. 2009; 71: 1239–1242. 10.1292/jvms.71.1239 19801907

[17] Soumyalekshmi S, Ajith MK, Meshram C. Isolation of fowl adenovirus in chicken embryo liver cell culture and its detection by hexon gene based PCR. Indian Journal of Scientific Research and Technology. 2014; 2(3): 33–36.

[18] Xie Z, Fadl A, Girshick T, Khan M. Detection of Avian Adenovirus by Polymerase Chain Reaction. Avian Dis. 1999; 43(1): 98–105. 1999. 10216765

[19] Meulemans G, Boschmans M, Berg TP, Decaesstecker M. Polymerase chain reaction combined with restriction enzyme analysis for detection and differentiation of fowl adenoviruses. Avian Pathol. 2001; 30:655–660. 10.1080/03079450120092143 19184959

[20] Raue R, Hess M. Hexon based PCRs combined with restriction enzyme analysis for rapid detection and differentiation of fowl adenovirus and egg drop syndrome virus. J. Virol. Methods. 1998; 73: 211–217. 10.1016/s0166-0934(98)00065-2 9766892

[21] Mouahid M, Bengoumi M, Kichou F. Case report of recent outbreak of inclusion body hepatitis among broilers in Morocco. Abstract. Proc. 18th World Veterinary Poultry Association Congress, Nantes, France. 2013; pp. 562.

[22] Said Anas. Etude clinico-pathologique de cas d’hépatite à inclusion chez la volaille entre 2012 et 2015. Thesis. Institut Agronomique et Vétérinaire Hassan II, Rabat, Morocco. 2015.

[23] Salek M, El Houadfi M. The first case and description of FAdV-11 causing high mortalities in very young broiler breeders in Morocco. Abstract. Proc. 19th World Veterinary Poultry Association Congress, Edinburgh, UK. 2017.

[24] Redondo H, Sarabia FJ, Ait Tahala M, Bensassi Y, Gil I, Elbachir Erraji, et al Characterization of strain of fowl adenoviruses circulating in Morocco. Poult Sci. 2018; 0:1–6. 10.3382/ps/pey271 29982730

[25] Schachner A, Matos M, Grafl B, Hess M. Fowl adenovirus-induced diseases and strategies for their control- a review on the current global situation. Avian pathol. 2018; 47(2): 111–126. 10.1080/03079457.2017.1385724 28950714

[26] Mendelson C, Nothelfer HB, Monreal G. Identification and characterization of an avian adenovirus isolated from a spiking mortality syndrome field outbreak in broilers on the Delmarva Peninsula, USA. Avian Pathol. 1995; 24:693–706. 10.1080/03079459508419108 18645825

[27] Mittal D, Jindal N, Tiwari AK, Khkhar RS. Characterization of fowl adenoviruses associated with hydropericardium syndrome and inclusion body hepatitis in broiler chickens. Virusdisease. 2014; 25:114–119. 10.1007/s13337-013-0183-7 24426318 PMC3889237

[28] Ojkic D, Martin E, Swinton J, Vaillancourt JP, Boulianne M, Gomis S. Genotyping of canadian isolates of fowl adenoviruses. Avian Pathol. 2008; 37:95–100. 10.1080/03079450701805324 18202956

[29] Kajan GL, Kecskemeti S, Harrach B, Benko M. Molecular typing of fowl adenoviruses, isolated in Hungary recently, reveals high diversity. Vet Microbiology. 2013; 167: 357–363. 10.1016/j.vetmic.2013.09.025 24139719

[30] Choi KS, Kye SJ, Kim JY, Jeon WJ, Lee EK, Park KY, et al Epidemiological investigation of outbreaks of fowl adenovirus infection in commercial chickens in Korea. Poult. Sci. 2012; 91:2502–2506. 10.3382/ps.2012-02296 22991534

[31] Shaib H, Ramadan N, Mahmoud G, Nassif G, Chedid S. Outbreak of inclusion body hepatitis causing Adenovirus in Lebanese Broiler flocks. EC Microbiology. 2017; 13.3:92–101.

[32] Oliver-Ferrando S, Dolz R, Calderón C, Valle R, Rivas R, Pérez M, et al Epidemiological and pathological investigation of fowl aviadenovirus serotypes 8b and 11 isolated from chickens with inclusion body hepatitis in Spain (2011–2013). Avian Pathol. 2017; 46(2): 157–165. 10.1080/03079457.2016.1232477 27928940

[33] Kaur G, Maiti NK, Oberoi M. Biological and immunological characterization of fowl adenovirus-4 isolates from inclusion body hepatitis-hydropericardium syndrome. Indian. J. Anim. Sci. 2003; 73(1):51–52.

[34] Kim JN, Byun SH, Kim MJ, Kim Jj, Sung HW, Mo IP. Outbreaks of hydropericardium syndrome and molec-ular characterization of Korean fowl adenoviral isolates. Avian Dis. 2008; 52:526–530. 10.1637/8178-112207-Case 18939647

[35] Kumar V, Kumar R, Chandra R, Bhatt P, Dhama K. Outbreaks of Inclusion Body Hepatitis (IBH) in Chickens; Pathological Studies and Isolation of Fowl Adenovirus. Advances in Animal and Veterinary Sciences. 2013; 1(3(S)):21–24.

[36] Almemnesc W, Hair-Bejo M, Aini I, Omar AR. Pathogenicity of fowl adenovirus in specific pathogen free chicken embryos. J Comp Pathol. 2012; 146: 223–229. 10.1016/j.jcpa.2011.05.001 21705014

[37] Matos M, Grafl B, Liebhart D, Hess M. The outcome of experimentally induced inclusion body hepatitis (IBH) by fowl aviadenoviruses (FAdVs) is crucially influenced by the genetic background of the host. J. Vet. Res. 2016; 47: 69. 10.1186/s13567-016-0350-0 PMC4928300 27356980

[38] Steer-Cope P, Sandy J, O’Rourke D, Scott P, Browwning G, Noormohammadi A. Chronologic analysis of gross and histologic lesions induced by field strains of FAdV-1, FAdV-8b, FAdV-11 in six week old chickens. Avian Dis. 2017; 61(4): 512–519. 10.1637/11718-072317-Reg.1 29337616

[39] Domanska-Blicharz K, Tomczyk G, Smietanka K, Kozaczynski W, Minta Z. Molecular characterization of fowl adenoviruses isolated from chickens with gizzard erosions. Poult Sci. 2011; 90:983–989. 10.3382/ps.2010-01214 21489943

[40] Niczyporuk JS. Molecular characterization of fowl adenovirus type 7 isolated from poultry associated with inclusion body hepatitis in Poland. Arch. Virol. 2017; 162:1325–1333. 10.1007/s00705-017-3240-5 28160143 PMC5387021

[41] Niczyporuk JS, Wozniakowski G, Samorek-Salamonowicz E. Application of cross-priming amplification (CPA) for detection of fowl adenovirus strain. Arch. Virol. 2015; 160:1005–1013. 10.1007/s00705-015-2355-9 25655263 PMC4369288

[42] Gulhane AB, Deshpande AA, Gogoi S, Kumar P. Isolation and characterization of different fowl adenovirus types associated with inclusion body hepatitis in broiler chickens of India. J Pure Appl Microbio. 2016; 10(1): 417–423.

[43] Absalon AE, Morales-Garzon A, Vera-Hernandez PF, Cortés-Espinosa DV, Uribe-Ochoa SM, Garcia LJ, et al Complete genome sequence of a non-pathogenic strain of fowl adenovirus serotyoe 11: Minimal genomic differences between pathogenic and non-pathogenic viruses. J Virol. 2016; 501: 63–69. 10.1016/j.virol.2016.11.006 27865971

[44] Asthana M, Singh VK, Kumar R, Chandra R. Isolation, cloning and silico study of hexon gene of fowl adenovirus 4 isolates associated with hydro pericardium syndrome in domestic fowl. J Proteomics Bioinform. 2011; 4(9): 190–195.

[45] Dahiya S, Srivastava RN, Hess M, Gulati BR. Fowl Adenovirus serotype4 Associated with Outbreaks of Infectious Hydropericardium in Haryana, India. Avian Dis. 2002; 46:230–233. 10.1637/0005-2086(2002)046[0230:FASAWO]2.0.CO;2 11922341

[46] Grafl B, Liebhart D, Gunes A, Wernsdorf P, Aigner F, Bachmeier J, et al Quantity of virulent fowl adenovirus serotype 1 correlates with clinical signs, macroscopical and pathohistological lesions in gizzards following experimental induction of gizzard erosion in broilers. Vet Res. 2013; 44:38 10.1186/1297-9716-44-38 23705834 PMC3672026

[47] Hong-Su P, II-Soo L, Sang-Kyu K, Toh-Kyung K, Sang-Geon Y. Isolation and characterization of fowl adenovirus serotype 4 from chicken with hydropericardium syndrome in korea. Korean. J. Vet. Res. 2001; 51(3): 209–216.

[48] Radwan MM, El-Deeb AH, Mousa MR, El-Sanousi AA, Shalaby MA. First report of fowl adenovirus 8a from commercial broiler chickens in Egypt: molecular characterization and pathogenicity. Poult Sci. 2018; 0:1–8. 10.3382/ps/pey314 30690614

[49] Mase M, Nakamura K, Minami F. Fowl adenoviruses isolated from chickens with inclusion body hepatitis in Japan, 2009–2010. J. Vet. Med. Sci. 2012; 74(8):1087–1089. 10.1292/jvms.11-0443 22516693

[50] Niczyporuk JS, Samorek-Salamonowicz E, Czekaj H. Analysis of adenovirus strains isolated from poultry in Poland. Bull Vet Inst Pulawy. 2013; 57: 305–310.

[51] Okuda Y, Ono M, Shibata I, Sato S. Pathogenicity of serotype 8 fowl adenovirus isolated from gizzard erosions of slaughtered broiler chickens. J. Vet. Med. Sci. 2004; 66:1561–1566. 10.1292/jvms.66.1561 15644608

[52] Philippe C, Grgic H, Ojkic D, Nagy É. Serologic monitoring of a broiler breeder flock previously affected by inclusion body hepatitis and testing of the progeny for vertical transmission of fowl adenoviruses. Can. J. Vet. Res. 2007; 71:98–102. 17479772 PMC1829188

[53] Dar A, Gomis S, Shirley I, Mutwiri G, Brownlie R, Potter A, et al Pathotypic and Molecular characterization of a fowl adenovirus associated with inclusion body hepatitis in Saskatchewan chickens. Avian Dis. 2012; 56:73–81. 10.1637/9764-041911-Reg.1 22545531

[54] Ojkic D, Krell PJ, Tuboly T, Nagy É. Characterization of fowl adenoviruses isolated in Ontario and Quebec, Canada. Can. J. Vet. Res. 2008; 72:236–241. 18505186 PMC2327250

[55] Hemida MG, Al-Hammadi M. Prevalence and molecular charactestics of fowl adenovirus serotype 4 in eastern Saudi Arabia. Turk. J. Vet. Anim. Sci. 2017; 41:506–513.

[56] Ganesh K, Suryanarayana VVS, Raghavan R. Detection of fowl adenovirus associated with hydropericardium hepatitis syndrome by a polymerase chain reaction. Vet.Res.Commun. 2002; 26:73–80. 10.1023/a:1013361906791 11862998

[57] Rahul S, Kataria JM, Senthilkumar N, Dhama K, Uma R, Sylvester SA, et al Polymerase chain reaction based differentiation of various fowl adeovirus serotypes causing inclusion body hepatitis-hydropericardium syndrome of poultry in india. Indian. J. Comp. Immunol. Microbiol. Infect. Dis. 2004; 25(1):1–6.

[58] Mase M, Nakamura K. Phylogenetic analysis of fowl adenoviruses isolated from chickens with gizzard erosion in Japan. J. Vet. Med. Sci. 2014; 76(11):1535–1538. 10.1292/jvms.14-0312 25131809 PMC4272990

[59] Zhao J, Zhong Q, Zhao Y, Hu Y X, Zhang G Z. Pathogenicity and Complete Genome Characterization of Fowl Adenoviruses Isolated from Chickens Associated with Inclusion Body Hepatitis and Hydropericardium Syndrome in China. Plos one. 2015; 10(7): e0133073 10.1371/journal.pone.0133073 26167857 PMC4500579

[60] Ghafari PT, Boroomand Z, Rezaie A, Mayahi M, Eftekharian S. Detection and identification of avian adenovirus in broiler chickens suspected of inclusion body hepatitis in Khuzestan, Iran during 2015–2016. Iranian Journal of Veterinary Science and Technology. 2018; 9(2): 41–45.

